# A comparison of treatment response between college students with and without suicide risk at university counseling centers

**DOI:** 10.1080/28324765.2023.2169583

**Published:** 2023-04-17

**Authors:** Bethany A. Rallis, Justin Petrovich, Brett E. Scofield, Sangsun Kim, Benjamin D. Locke

**Affiliations:** a Department of Student Affairs, Virginia Polytechnic Institute and State University, Blacksburg, VA, USA; b Department of Statistics and Business Analytics, Saint Vincent College, Latrobe, PA, USA; c Department of Student Affairs, Pennsylvania State University, State College, PA, USA; d Department of Student Affairs, University of Florida Gainesville, FL, USA

**Keywords:** Suicidal ideation, suicide attempt, suicide risk, university counseling centers, treatment

## Abstract

This study aims to explore the effect of University Counseling Center (UCC) treatment in a nationally representative sample of 101,354 college students with suicide risk variables (i.e. recent suicidal ideation (SI), recent SI and history of suicide attempts (SA), history of SA) as compared to those without suicide risk seeking services from 160 UCCs. Time Varying Effects Modeling was used to assess changes in distress level and SI over the course of up to 20 sessions of treatment among students with the aforementioned risk variables compared to those without. Reductions in distress and SI were observed in all students with the most significant change seen in the first five sessions. Students with recent SI and no history of attempts seem to achieve comparable amount of improvement to students without suicide risks. Presence of suicide attempt history appears to limit the prognosis. These results entail that students with a history of SA whose levels of risk and chronicity of symptoms may require more treatment and be better managed with alternative forms of treatment.

## Clinical or methodological significance

Service utilization, especially among suicidal students, has increased to the point of potentially exceeding the capacity of UCCs to provide effective treatment. Research on treatment response customarily uses methods that constrain the treatment curve. This study uses methods that capture the actual treatment curve as it may vary based on mode of treatment and level of severity of the presenting concern. These methods offer an investigation, without constraints to the treatment curve, into the effects of treatment on students presenting with suicide risk that may inform UCC policy and session limits.

The suicide rate for individuals ages 15 to 24 has been gradually increasing since 2010 (Centers for Disease Control, 2021). In fact, suicide has been the second leading cause of death among college students for over a decade (Turner et al., 2013; Suicide Prevention Resource Center [Suicide Prevention Resource Center, 2004) and accounts for approximately 1,100 deaths per year on college campuses (Appelbaum, 2006). Suicidal ideation (SI) severity (i.e. intent, method) and intensity (i.e. frequency, controllability), as well as a lifetime history of suicide attempt (SA) are strong predictors of future suicide attempts within the college population (Horowitz, A.G., Czyz, E.K. & King, C.A, 2014). Nationally, 13% of college students in the general population seriously considered suicide in the past year and 1% made a suicide attempt (Healthy Minds Study, Winter 2021). Among students seeking mental health services, 22% seriously considered attempting suicide and 13% made a suicide attempt in the past year (Center for Collegiate Mental Health (CCMH), 2023).

Meanwhile, the demand for counseling services among college students has been increasing for decades. In fact, from 2009 to 2015, the growth in the number of students seeking services at counseling centers (+29.6%) was more than 5x the rate of institutional enrollment (+5.6%; Center for Collegiate Mental Health (CCMH), 2015). This increase in demand was disproportionately represented by students with lifetime serious consideration of suicide, increasing from 30.1% of students in 2012 to 34.2% of students in 2022 (Center for Collegiate Mental Health (CCMH), 2023). Moreover, students with histories of self-harm/suicidal thoughts and behaviors consumed, on average, 27% more services than those without these characteristics (Center for Collegiate Mental Health (CCMH), 2016).

One of the negative consequences of the perpetual supply-demand imbalances occurring within UCCs nationally is rising counselor caseloads (Center for Collegiate Mental Health (CCMH), 2020). From 2019 to 2021, CCMH demonstrated that higher annual caseloads of clinicians were associated with diminished treatment effectiveness (less improvement in symptoms) and reduced treatment dose, which impacts all students, on average, including those with critical safety issues and identity concerns. Thus, even students with SI, who would be considered in critical need of services, received less treatment at UCCs where counselors have high versus low caseloads. An important question to consider in suicide prevention on college campuses is whether UCCs are able to effectively accommodate the increasing requests for services with the current resources available, especially among students presenting with suicide risk. In sum, the pervasive dilemma encountered by UCCs, where demand for services frequently outpaces the supply of treatment available, underscores the significance of evaluating the dosage of treatment needed to be effective in serving students with high levels of suicide risk who are utilizing an increasing portion of the services offered at UCCs nationally.

Two approaches to understanding how much therapy is required for improvement are the Dose Effect (DE) and Good-Enough-Level (GEL). According to the DE model, improvement in therapy is related to the dosage of treatment (e.g. the number of sessions attended), typically with the caveat that earlier sessions have stronger effects than later (Falkenström et al., 2016). That is, dosage determines effect, but with diminishing returns. The GEL model dismisses the notion that dosage determines improvement and instead assumes that the benefit of therapy is commensurate with a client’s idiosyncratic needs, so that clients attend therapy until they reach their own “good enough” level. Each client’s rate of change may vary based on variables such as presentation of symptoms (Barkham et al., 2006; Barkham et al., 1996; Owen et al., 2016; Stiles et al., 2008) and frequency of sessions (i.e. more sessions on average per week leads to more rapid improvement; Reese et al., 2011). Studies suggest that improvement is slower for chronic and acute concerns (Kopta et al., 1994; Niileksela et al., 2021). Thus, students presenting with more acute and chronic presentations may improve more slowly and require a greater number of sessions at UCCs.

UCCs, like other naturalistic settings, generally implement a short-term treatment model by staff who are heterogeneous in their theoretical orientation and approach to treatment (Center for Collegiate Mental Health (CCMH), 2013). In general, treatment in a UCC setting was effective in producing reductions in distress and symptom change across a wide range of diagnoses, including major depressive disorder, generalized anxiety disorder, social phobia, and eating disorders (Center for Collegiate Mental Health (CCMH), 2017; Østergård et al., 2019; A.A. McAleavey et al., 2019, Minami et al., 2009; Murray et al., 2016). However, the outcomes, on average, did not result in a return to normative functioning for those students who initiate treatment with more severe difficulties (A.A. McAleavey et al., 2019). This information raises important questions about the optimal treatment dosages needed to produce reliable change for individuals experiencing more critical issues, particularly those with suicide risk who initiate services at UCCs. In particular, research is needed to determine what dosage of treatment is optimal for students with high suicide risk.

Prior research suggests that the treatment dosage for students with suicide risk varies depending on the modality and level of risk (i.e. current SI with no history of suicide attempts compared to those chronic SI and a history of attempts). Collaborative Assessment and Management of Suicidality (CAMS; Jobes, 2006, 2016) and Dialectical Behavior therapy (DBT; Linehan, 1993; Linehan et al., 2006) are the primary treatments demonstrating effectiveness at addressing suicide risk among college students in UCCs (Jobes et al., 1997; Pristorello et al., 2012). CAMS showed promise treating students with less severe presentation of suicide risk (e.g. with no history of suicide attempts; Pistorello et al., 2020) in as few as four to eight sessions (see Pistorello et al., 2017). Whereas DBT has demonstrated effectiveness at reducing suicidality in 7-12 months and among students with more chronic symptoms (Pistorello et al., 2012).

Although many counseling centers implement a short-term model of care, students who enter services with heightened levels of suicide risk frequently require longer-term care beyond the typical routine student who averages approximately 4.5 counseling sessions (Center for Collegiate Mental Health (CCMH), 2017). In fact, students with histories of serious suicidal ideation and attempts use approximately 25% to 30% more services than those without these attributes (Center for Collegiate Mental Health (CCMH), 2016). National UCC data suggest that students, on average, experience symptom reduction during services at various rates, and individuals with chronic needs might eventually experience an equivalent improvement from treatment than those who change more quickly if they are afforded longer-term care and extended doses of treatment (Center for Collegiate Mental Health (CCMH), 2017).

Conventionally, research on dose-response relationships has utilized multilevel growth curve models, which often use a log normal curve to explicitly model session-to-session change during therapy according to a predicted model (e.g. Lutz et al., 2001; Lutz et al., 1999). While a logarithmic curve may suitably describe the improvement or deterioration of some client groups, it is also possible that other groups may exhibit different patterns of change throughout treatment. Thus, it is important to use statistical models that allow the course of treatment to demonstrate the average treatment response curve as it may vary based on mode of treatment and level of severity of the presenting concern. One such model is the Time Varying Effect Model (TVEM; Tan et al., 2012). In short, the primary advantage of TVEM over other statistical strategies for estimating time-varying effects is the flexibility it offers to capture nonlinear patterns with minimally restrictive assumptions.

For instance, when studying the change in student distress throughout the course of treatment, both time-varying and time-invariant independent variables may be used to explain the dependent variable, distress. If differences between students with and without recent suicidal ideation at intake are of interest, this can be studied by including an indicator of SI as an independent variable with a time-varying effect. A priori it is difficult to know what pattern of improvement will be exhibited by either of these groups, but a TVEM allows one to leave this unspecified (e.g. not to predetermine that the change must follow a logarithmic trend). Instead, the time-varying effect of initial suicidal ideation on student distress throughout treatment is determined by the data over time. Thus, if student distress tends to improve at a non-constant rate throughout treatment, or if an even more complicated nonlinear pattern of improvement emerges, TVEM can capture it.

The dose-response relationship of routine treatment offered to students presenting with different suicide risk variables at UCCs has received little attention and has never been analyzed using TVEM. Research on the effectiveness of routine treatment using naturalistic data and methodology that allows the course of treatment to define the change curve is particularly important given that UCC treatment (i.e. therapeutic approaches and length of treatment) provided to address suicide risk varies based on therapist choice of treatment approach and is limited given the capacity of UCCs to serve the growing number of students presenting for care. Thus, research designed to investigate the effects of the heterogeneous treatment provided to students presenting with suicidality across UCCs is critical to suicide prevention efforts. Specifically, insight into the dose-response relationship in the care of students presenting to UCCs with these risk factors will inform practices and recommendations.

### Present study

The present study aims to address gaps evident in suicide treatment literature on college campuses by initiating preliminary exploration of treatment trajectories utilizing TVEM to analyze treatment response in naturalistic data from a nationally representative sample of students presenting to UCCs with suicidality. More specifically, we are interested in how levels of distress (as a global indicator of treatment effects) and SI change over the course of treatment and how that pattern of change differs between groups of students who do and do not present to their first session of treatment with suicide risk variables. To draw comparison between groups of students with varying degrees of risk, the four groups of students were divided according to: (1) those with historical attempts but no recent SI, (2) those with recent SI but no historical attempts, (3) those with both historical attempts and recent SI, and (4) those with neither recent SI nor historical attempts based on SDS. To study this, two separate TVEMs were used: one to model change in distress and the other to model change in SI over the course of treatment. In both models, indicators of the suicide risk variables were used as independent variables and their time-varying effects are of primary interest.

Consistent with the DE model, it was hypothesized that overall routine treatment would be associated with decreases in distress and SI among students who reported SI in the past 2 weeks with no history of SA and among students who denied suicidal ideation within the past 2 weeks but endorsed history of SA. Consistent with the GEL models, it was also hypothesized that, while students reporting history of SA and current SI at their initial appointment will likely demonstrate decreased distress, their change in distress level may happen more slowly (i.e. require more sessions for improvement). These hypotheses will be tested for all students in the sample and also within a subsample of only those students who began with a high level of distress. Analyzing an isolated sample of students with a high level of distress allows us to examine clients with an identified problem and improves sensitivity to change in the analyses (A.A. McAleavey et al., 2019).

## Method

### Procedures

The current study utilized standardized data collected from members of Center for Collegiate Mental Health (CCMH), a Practice-Research-Network, via two instruments described below under measures. All UCCs participating in CCMH secure and maintain their own Institutional Review Board approval. CCMH members download their local de-identified data from students, presenting at UCCs as a part of their routine treatment, to CCMH’s centralized data repository once a year (McAleavey et al., 2015). For the present study, researchers collected an anonymized and aggregated data set from CCMH to conduct the analyses described below.

### Participants

The current study used data gathered by CCMH members across five academic years (2012-2017). The combined data set in this study includes a nationally representative sample of 101,354 students seeking services from 160 UCC members of CCMH. The total sample denotes the number of unique first courses of treatment for students included across the five academic years, where a new course of treatment is defined when there are more than 90 days between consecutive appointments. Prior to analyses, the data set was reduced to include only first course of therapy for each client. This means that students were included for only one course of treatment across the five years even if they participated in multiple episodes of treatments. Thus, students are not represented in multiple years if they returned for courses of treatment in different years. In addition, only attended individual counseling treatment appointments were included. The data set was also limited to students with at least two Counseling Center Assessment of Psychological Symptoms (CCAPS) administrations (CCAPS 62 and 34; see Measures section for further information) and students who completed the CCAPS within 14 days of their first appointment. Finally, to be included in the study, students were required to have responded to an additional set of questions assessing for suicidality on a separate measure.

Important to the analysis in this study is the number of appointments attended by clients. While the data included students who attended well over 100 sessions, such students were rare. In order to ensure a reasonable level of precision for statistical analysis of change per session, a large enough sample of students was needed to attend each particular number of sessions. Therefore, only the first 20 sessions for any client were retained for further analysis. Though any imposed cutoff would be somewhat subjective, there were still 2,186 students who attended 20 sessions. Higher session numbers than 20 corresponded to successively fewer students, which in turn would diminish statistical precision when analyzing changes for those session numbers. After imposing the 20-session cutoff for analysis purposes, the average number of sessions attended per client was 7.1 (SD = 4.6). Table 1 summarizes the distribution of sessions attended and shows that across each of the four sub-samples of interest, the number of sessions attended was very similar.

**Table 1.** Summary statistics for number of sessions attended by subsample.

		Number of Sessions Attended						
Sub-sample	*N*	Min	Quartile 1	Median	Mean	Quartile 3	Max	SD
noSI_noSA	87,945	1.0	4.0	6.0	7.0	9.0	20.0	4.6
noSI_SA	8,006	1.0	4.0	6.0	7.5	10.0	20.0	4.9
SI_noSA	3,805	1.0	4.0	6.0	7.4	10.0	20.0	4.8
SI_SA	1,598	1.0	4.0	6.0	7.4	10.0	20.0	4.9
Total	101,354	1.0	4.0	6.0	7.1	9.0	20.0	4.6

The average age of participants was 22.3 years (SD = 4.5). The sample contained a majority of women (63.9%), followed by men (31.5%), those choosing to self-identify or not respond (4.1%), and about 0.5% identifying as transgender. The majority of the sample identified as White (67.5%), followed by Hispanic (8.5%), African American (8.0%), Asian American (6.2%), and multi-racial (4.5%) with about 5.4% reporting as Native American, Arab American, Hawaiian/Pacific Islander, other ethnicity, preferred to self-identify, or chose not to answer the question. The majority of students identified as heterosexual (77.2%), followed by bisexual (6.4%), gay or lesbian (4.5%), self-identified (3.1%), questioning (2.5%), and 6.4% chose not to answer this question. With regard to student status: 19.7% identified as first-year, 18.8% as second-year, 22.0% as third-year, 19.5% as fourth-year, and 20.0% as other. Of the 101,365 clients, 87,945 (86.8%) had neither a history of suicide attempts nor acute suicidality, 8,006 (7.9%) identified a history of suicide attempts but no acute suicidality, 3,805 (3.8%) identified acute suicidality but no history of suicide attempts, and 1,598 (1.6%) indicated both a history of suicide attempts and acute suicidality.

### Measures

#### Standardized data set

The standardized data set (SDS) is a set of standardized questions designed to collect information about client demographics and mental health history. UCCs typically administer the SDS pretreatment (Hayes et al., 2011). However, timing of administration may vary based on the UCC policy. The SDS items assess for both recency (“the last time”) and frequency (“how many times”). The following two items were selected from the mental health history section of the SDS for the current study: “Seriously considered attempting suicide”; “made a suicide attempt”. These items ask how many times an event has occurred in a client’s life, including “never”, 1 time, 2-3 times, 4-5 times, and “more than 5 times”, and when the event occurred last from “never”, “within the last 2 weeks”, “within the last month”, “within the last year”, “within the last 1-5 years”, “more than 5 years ago”. Responses were simplified for the present analyses to identify whether students endorsed having SI within the last 2 weeks (prior to being administered the SDS) and whether they endorsed ever having made a suicide attempt in their lifetime. From there, students were divided into four groups based on their report of current SI and history of SA: 1) SI in the past two weeks before their first appointment with no history of suicide attempts (SI_noSA); 2) SI in the past two weeks with a history of SA (SI_SA); 3) history of SA with no SI in the past two weeks (noSI_SA); and 4) no SI in the past two weeks and no history of SA (noSI_noSA).

#### Counseling center assessment of psychological symptoms

The Counseling Center Assessment of Psychological Symptoms (CCAPS; Locke et al., 2011;) is a multidimensional measure of psychological symptoms designed specifically to assess college mental health difficulties, and it is administered pre-, during, and post-treatment, depending on center policy. The CCAPS-34 includes 34 items which ask students to rate how well the items describe them in the past two weeks on a five-point Likert scale ranging from 0 (not at all like me) to 4 (extremely like me). The CCAPS-34 provides seven factor analytically derived subscales for Depression, Generalized Anxiety, Social Anxiety, Eating Concerns, Alcohol Use, Academic Distress, and Hostility (Locke et al., 2011). Twenty items drawn from these subscales comprise the Distress Index, an additional subscale of the CCAPS-34 (McAleavey et al., 2012). In addition, the CCAPS contains an item (item #25) to assess SI which asks participants to rate the prompt “I have thoughts of ending my life” (in the last two weeks) on the Likert scale described above. The SI item was used as a dependent variable and will hereafter be referred to as CCAPS SI to differentiate it from the SDS SI item, which acts as an independent variable. The SDS SI item will be referred to as SDS SI throughout the rest of the paper. The measure has demonstrated strong convergent validity with other established measures of related domain-specific distress, and appropriately low correlations with unrelated domains (Locke et al., 2011; McAleavey et al., 2012). The 1- and 2-week test-retest reliability in nonclinical samples ranges from 0.76 for Academic Distress to 0.92 for Depression (McAleavey et al., 2012). The internal consistency for the current study, as measured by Cronbach’s alpha, was 0.92.

Of note, the CCAPS provides cut scores for each subscale. CCAPS subscale cut scores divide each subscale into ranges of low, moderate, and high distress in a given subscale (McAleavey et al., 2012). CCAPS subscale scores that fall below the high cut represent students who report moderate to no, or minimal distress, whereas scores above the high cut are consistent with high enough levels of distress on a given subscale as to warrant further assessment for a diagnosis in the diagnostic area associated with the subscale (i.e. likelihood of a diagnosis increases as the score increases above the high cut).

### Data analysis

#### Time varying effects modeling

Prior to selecting a statistical model for these data, preliminary exploratory analyses were performed and can be found in the Supplementary Material. In addition to the goal of studying relationships between client factors and distress throughout treatment, two important features of the data to consider for statistical modeling were the longitudinal nature of the data and the potential correlation between individual clients who saw the same therapist. More specifically, since some clients saw multiple therapists, these factors were partially crossed. While multilevel models (MLMs) can effectively model these kinds of data, we instead chose to employ a Time Varying Effect Model.

An extension of linear regression that can capture time-varying effects of either time-varying or time-invariant covariates, TVEMs model changes in the associations between the independent variables (i.e. suicide risk groups) and the dependent variable (i.e. distress, CCAPS SI) over time (i.e. the course of treatment at UCCs) without requiring a pre-specified change pattern or division of time into arbitrary intervals. Whereas MLMs require a parametric (e.g. logarithmic, quadratic, or other polynomial) change pattern that is assumed known a priori, TVEMs’ strength lies in their ability to nonparametrically estimate the shape of smooth, potentially nonlinear time-varying effects. This allows greater flexibility in modeling by freeing researchers from the restrictive burden of specifying a parametric relationship a priori. In practice, TVEMs can be especially advantageous as a first modeling technique to assess the plausibility of a parametric relationship, or for modeling complex nonlinear patterns that aren’t easily captured by logarithmic or other parametric forms. For a further comparison of TVEMs to MLMs and latent growth curve models, see, Wright et al. (2014).

We employed two different time-varying effect models in our analyses, a linear TVEM using DI as the time-varying dependent variable and an ordinal TVEM using CCAPS SI as the time-varying dependent variable. In both models we used the same independent variables, SDS SI and SA, to study their relationship with the dependent variable across sessions of treatment. Since there was considerable variability in terms of the number of sessions that clients attended and since this variability could plausibly confound the effects of interest, we additionally included the number of attended sessions as time-varying independent variables in each model. In the literature on client change trajectories this is consistent with the good-enough-level (GEL) model (Baldwin et al. 2009; Falkenström et al., 2016; Owen et al., 2016). We found that the effect of number of sessions was negligible in all cases, so we report results here from a re-analysis that omitted this variable. The results of the models that included number of attended sessions as a control variable are shared in the Supplementary Material.

Using the binary indicators of suicidal ideation within the past 2 weeks (SDS SI) and history of suicide attempts (SA) as covariates, the model to predict distress index (DI) takes the following form:

(1) $$DI_{ij}\left(t_{ij}\right)=\beta_{0}\left(t_{ij}\right)+\beta_{1}\left(t_{ij}\right)SI_{i}+\beta_{2}\left(t_{ij}\right)SA_{i}+b_{i}\left(t_{ij}\right)+\varepsilon_{ij}\left(t_{ij}\right),$$

where i indexes the individual client and j indexes the session of therapy, so that $t_{ij}$ represents the $j^{th}$ appointment for the $i^{th}$ client. The errors, $\varepsilon_{ij}$, are assumed to be independent and identically distributed across clients and measurements such that $\varepsilon_{ij}∼N\left(0,\sigma_{\varepsilon }^{2}\right)$. The $b_{i}$ are functional random intercepts assumed to be mean-zero Gaussian processes (Scheipl et al., 2015). Similar in spirit to the more conventional random intercepts used in mixed or multilevel models, these account for client-specific differences in distress but also allow for these differences to change over the course of treatment (i.e. the client-specific differences are treated as a function of appointment number). Ideally, the model would include a random intercept term for therapist-specific differences in distress change, and possibly random slopes for both clients and therapists as well, but these more complicated random effects structures would add to the complexity of the model and software currently prohibits this for the kind of longitudinal data we have.^1^

Finally, in contrast to classical linear regression, the coefficients $\beta_{0}$, $\beta_{1}$, and $\beta_{2}$ are assumed to be smooth functions—in this case, functions of session number. Still, the coefficient functions can be interpreted in a manner that is a straightforward extension of the usual linear regression coefficients. That is, $\beta_{0}\left(t_{ij}\right)$ represents the mean level of DI for client i at appointment j if client i endorses neither SI nor SA. $\beta_{1}\left(t_{ij}\right)$, then, represents the shift in mean level of DI at appointment j for a client i who endorses SI, holding SA constant. Similarly, $\beta_{2}\left(t_{ij}\right)$ is the shift in mean level of DI at appointment j for a client i who endorses SA, holding SDS SI constant. Since these coefficients are functions, they are more commonly interpreted visually, by plotting the entire estimated function over the domain of interest. We will refer to this as Model (1).

In addition to client distress, changes in SI over the course of treatment can inform the treatment effectiveness. However, a model using the CCAPS SI item to estimate SI as a dependent variable does not fit into the framework of Model (1) since the CCAPS SI item is an ordinal variable. Rather, such a model requires the use of a generalized TVEM (Shiyko et al., 2014), an extension of TVEM to accommodate non-normal errors/response variables. Of particular interest, Dziak et al. (2014) introduced what they called ordinal TVEM—a proportional odds model allowing for time-varying effects of covariates on an ordinal response. Adapting their framework, we assumed that the level of SI of individual i at appointment j was truly a continuous but latent variable, $S_{ij}^{*}$, defined by

(2) $$S_{ij}^{*}\left(t_{ij}\right)=\beta_{0}\left(t_{ij}\right)+\beta_{1}\left(t_{ij}\right)SI_{i}+\beta_{2}\left(t_{ij}\right)SA_{i}+b_{i}\left(t_{ij}\right)+\varepsilon_{ij}\left(t_{ij}\right),$$

where the $\varepsilon_{ij}$ are independently and identically distributed according to the standard logistic distribution and the $b_{i}$ are student-specific functional random effects (again assumed to be mean-zero Gaussian processes). Note that this is slightly different from the model used by Dziak et al. (2014), where scalar random effects were employed rather than functional random effects. Since the level of SI, $S_{ij}^{*}$, is latent, what is instead observed is the proxy, CCAPS SI, which we will denote $S_{ij}$ and is determined by different thresholds of the latent variable. Thus, when $S_{ij}^{*}\leq c_{0}$, we observe $S_{ij}=0$, when $c_{0}<S_{ij}^{*}\leq c_{1}$ we observe $S_{ij}=1$, when $c_{1}<S_{ij}^{*}\leq c_{2}$ we observe $S_{ij}=2$, when $c_{2}<S_{ij}^{*}\leq c_{3}$ we observe $S_{ij}=3$, and when $c_{3}<S_{ij}^{*}$, we observe $S_{ij}=4$. Here, $c_{0}<c_{1}<c_{2}<c_{3}$ are the thresholds, or cut points, of the true (latent) SI which determine the observed response to CCAPS SI. Finally, the model can be written using the logit function as

$$logit\left(P\left(S_{ij}\leq k\right)\right)=logit\left(P\left(S_{ij}^{*}\leq c_{k}\right)\right)=c_{k}-\left[\beta_{0}\left(t_{ij}\right)+\beta_{1}\left(t_{ij}\right)SI_{i}+\beta_{2}\left(t_{ij}\right)SA_{i}+b_{i}\left(t_{ij}\right)\right],$$

for k=0,1,2,3.

For the analyses in this study, each model was fit via the computing software R using the pffr function within the refund package (Ivanescu et al., 2015). The pffr function estimates $\beta_{0}\left(t\right)$, $\beta_{1}\left(t\right)$, and $\beta_{2}\left(t\right)$ using penalized splines. Conceptually, this approach involves approximating each coefficient function via a spline basis expansion where the user must choose the number of basic functions; a roughness penalty is then applied to enforce smooth estimates. Ivanescu et al. (2015) describe the estimation methodology in greater detail. For our analyses, we used B-spline bases with a first-order difference penalty and the package’s default fast restricted maximum likelihood (f-REML) method for choosing the appropriate penalty parameters. In practice the precise number of basic functions is not crucial; typically, it is recommended to choose a basis dimension that is large enough to capture the full complexity of the coefficient function(s) and then let the penalty impose smoothness to avoid over-fitting the data. We used 10 basis functions to model the functional response variable and 10 basis functions to model the functional intercept. Increasing the number of basic functions beyond 10 did not change the overall shape of the estimated coefficients in any way other than by making them rougher.

## Results

Models (1) and (2) were each applied to two different samples, resulting in four model estimates. Students missing data on the response variable were left out of the respective model, so each model is based on a different sample size. The sample sizes used for each model are reported in Table 2, along with the proportion of the deviance explained by the estimated model. The results from these models are summarized corresponding to the hypotheses outlined above.

**Table 2.** Sample sizes for each model and the proportion of the deviance explained by the estimated model.

Sample	Model	N (number of students)	Deviance Explained
All	Linear	101,354	9.66%
	Ordinal	101,351	7.45%
Above High Cut	Linear	34,746	22.90%
	Ordinal	34,728	7.94%

### Change from routine treatment – all students

The first hypothesis tested was that treatment would improve the levels of distress and SI for both students reporting SI in the past 2 weeks and those with a history of SA. This hypothesis was first considered for the general population of students (i.e. CCAPS scores both above and below the high cut), so the entire sample of 101,354 clients was used. Based on the results from estimating the respective TVEM models, this hypothesis was found to be true on average for the groups of interest as well as for the students who reported both SI in the past 2 weeks and a history of SA, and for the students who reported neither of these. In other words, for all four groups of students, treatment corresponded with some improvement in their average levels of both distress and suicidal ideation.

**Figure 1.**
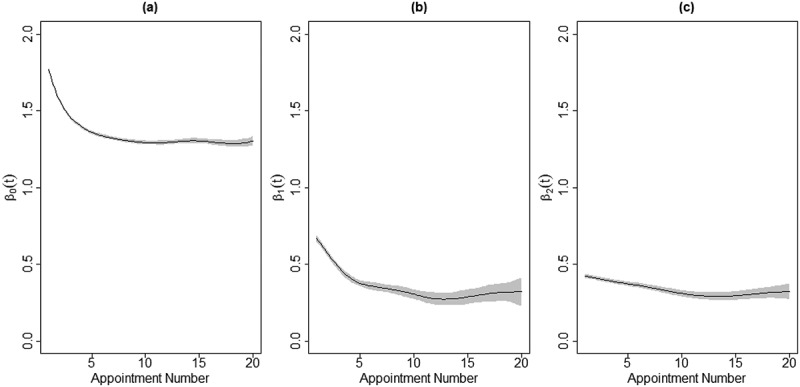
The estimated coefficient functions for the time-varying effects of SDS SI and SA on client distress

*Note*. the intercept coefficient (a), the coefficient for SI (b), and the coefficient for SA (c)

Model (1) was fit to assess the time-varying effects of SDS SI and SA on client distress. Since the estimated coefficients of the more commonplace linear regression models are replaced by estimated functions here, we report the results of our model visually. Specifically, Figure 1 shows the estimated coefficient functions: the intercept coefficient (a), the coefficient for SI (b), and the coefficient for SA (c). In each panel, the black line represents the estimated function and the gray shading represents pointwise 95% confidence bands around the function.

The intercept coefficient function, $\beta_{0}\left(t\right)$, shows the average DI at each appointment, t, among all clients in the noSI_noSA Group, when there is no random effect. For example, Figure 1(a) shows that the average DI level among noSI_noSA Group clients is around 1.8 at the start of treatment but declines sharply to around 1.3 by the seventh appointment, an indication of effective treatment. Unfortunately, however, the average DI level remains around 1.3 for the rest of treatment up to the 20^th^ appointment.

The coefficient function $\beta_{1}\left(t\right)$ represents the average change in DI at each appointment for clients with SDS SI compared to those without. Unsurprisingly, clients with SDS SI can expect to begin treatment 0.7 points higher in terms of DI than those without SDS SI, on average. Encouragingly, much of this additional distress is alleviated within 10-12 appointments. Finally, $\beta_{2}\left(t\right)$ measures the average difference in DI at each appointment for clients with past SA versus those who have no prior attempts. Clients with SA have on average an additional 0.4 points of DI at the start of treatment compared to noSA clients. Again, treatment seems to remove some of the additional distress associated with past suicide attempts, but only about 0.1 points of DI over the first 10-12 appointments and little, if any, after that.

**Figure 2.**
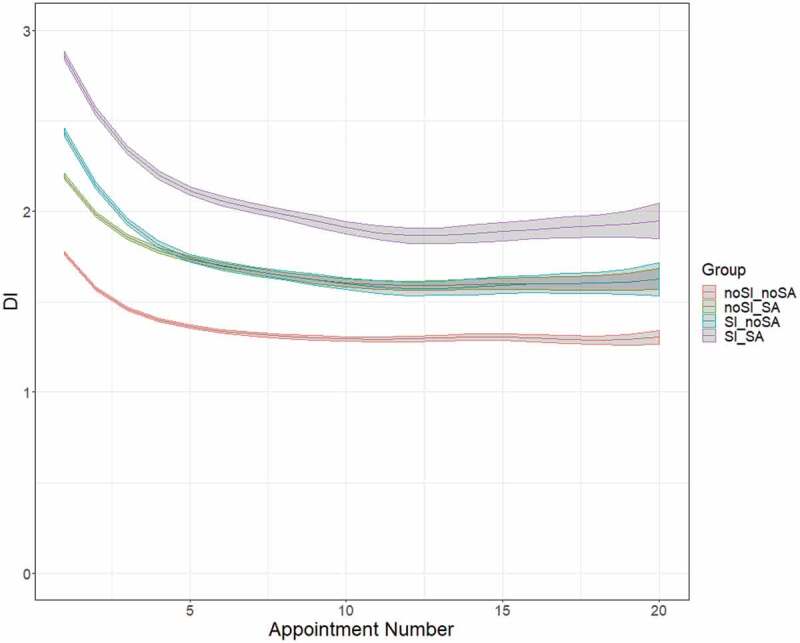
Predicted DI and the associated prediction bands for the four client groups

Figure 2 combines the information from each of these coefficients and shows predicted DI and the associated prediction bands for the four client groups of interest. It is clear from Figure 2 that the distress of each of the four groups improves, on average, with additional sessions of treatment, at least to a point. While clients in the SI_SA group tend to begin treatment with the highest distress, they also see the greatest improvement in distress over the course of treatment, seeing a full 1.0-point reduction in average DI after 12 appointments.

**Figure 3.**
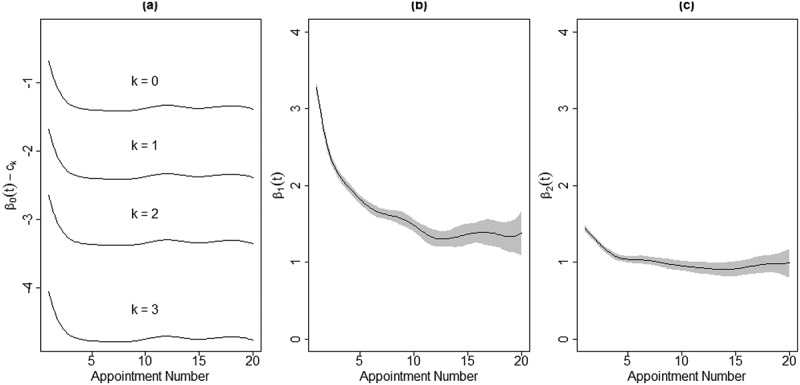
The estimated coefficient functions for the time-varying effects of SDS SI and SA on client CCAPS SI

Next, Model (2) was fit using the same data. Recall that the main difference from Model (1) is that in Model (2), the ordinal CCAPS SI item is used as the response (dependent) variable as opposed to DI, necessitating the use of a proportional odds model. The estimated coefficient functions are displayed in Figure 3. Instead of directly plotting the intercept coefficient function, $\beta_{0}\left(t\right)-c_{k}$ is shown because $\beta_{0}\left(t\right)$ alone is not identifiable. $\beta_{0}\left(t\right)-c_{k}$ can be interpreted as the log odds ratio of a CCAPS SI score greater than k among noSI_noSA Group clients with no random effect at appointment t. For instance, Figure 3 (a) shows that the estimated log odds ratio of a noSI_noSA Group client endorsing a CCAPS SI score greater than 3 (i.e. a 4) is about −4.1 at the first appointment and by the third appointment this estimated log odds ratio is about −4.7. These log odds ratios correspond with probabilities of 0.016 and 0.009, respectively. In other words, the probability of a noSI_noSA client endorsing a CCAPS SI score of 4 decreases from a 1.6% chance at the first appointment to a 0.9% chance by the third appointment, on average.

The slope coefficient functions, $\beta_{1}\left(t\right)$ and $\beta_{2}\left(t\right)$, have similar log odds interpretations. $\beta_{1}\left(t\right)$ represents the difference in log odds ratios of a higher CCAPS SI score between clients with SDS SI versus those without SI at appointment t, holding all else constant. In other words, higher values of $\beta_{1}\left(t\right)$ mean that students with SI are associated with a higher chance of reporting a larger CCAPS SI score. Similarly, $\beta_{2}\left(t\right)$ measures the difference in log odds ratios at appointment t of a higher CCAPS SI score between clients with SA versus those without SA, keeping other variables constant. Naturally, both SDS SI and SA increase the log odds ratio of a higher CCAPS SI score, though SDS SI to a much greater extent than SA. Importantly, both $\beta_{1}\left(t\right)$ and $\beta_{2}\left(t\right)$ are estimated to decrease with subsequent sessions. This indicates that the impact of SDS SI at the first appointment on CCAPS SI score diminishes throughout the course of treatment, as does the impact of SA on CCAPS SI score. Put another way, the relationships between these variables and CCAPS SI are improving as $\beta_{1}\left(t\right)$ and $\beta_{2}\left(t\right)$ shrink.

### Rate of change in distress – all students

The second hypothesis was that students reporting a history of SA and SI within the past 2 weeks at their first appointments would see a slower rate of improvement in their levels of distress, therefore requiring more sessions to see improvement. The results from estimating Model (1) for the entire sample of 101,354 clients don’t support this hypothesis. Contrary to the hypothesis, students reporting recent SI and a history of SA show moderately greater and more rapid improvement in distress than do students reporting either one or neither of these concerns at intake. Interestingly, those reporting recent SI (either with or without historical SA) realize larger and more rapid improvement in distress, on average, than do those reporting a history of SA. Furthermore, the greatest relief of distress for all four groups of students under study is realized within the first 5 sessions, while after the 12^th^ session, all groups cease to improve their average distress.

Focusing first on the rate of change in distress throughout treatment, Figure 2 is illuminating. Immediately evident from Figure 2 is the relatively rapid improvement in distress experienced across all groups within the first 2-3 appointments. Of note, though clients in the noSI_SA, SI_noSA, and SI_SA groups all start with average DI levels above the elevated DI threshold (2.15), after five appointments all of these clients experience enough improvement on average to drop below that elevated DI level. However, equally apparent is the deceleration of improvement in distress after around the 5^th^ appointment. For each group of clients, improvement in average DI tends to taper off entirely by the 12^th^ appointment.

### Change from routine treatment – high-distress students

In this section, the analyses described above for the first hypothesis were repeated on a subsample restricted to include only students who began treatment with CCAPS scores indicating a level of distress above the high cut (i.e. a high level of distress; DI greater than 2.15). The higher cut-point separates those with mild distress from those who are most likely to have a clinical concern in the relevant domain, in this case, distress (Center for Collegiate Mental Health (CCMH), 2013). Thus, refitting Models (1) and (2) on this targeted subsample allows us to focus analysis on clients who entered treatment highly distressed.

It was hypothesized that, even for this subsample, treatment would improve the levels of distress and SI for both students reporting SI in the past 2 weeks and those with a history of SA. This hypothesis was only partially confirmed, as treatment was correlated with declining distress among those reporting SI in the past 2 weeks. In contrast, those with a history of SA reported slightly higher distress, on average, with additional treatment. Both those with recent SI and past SA did show signs of improved SI over the course of treatment, however, supporting the hypothesis.

**Figure 4.**
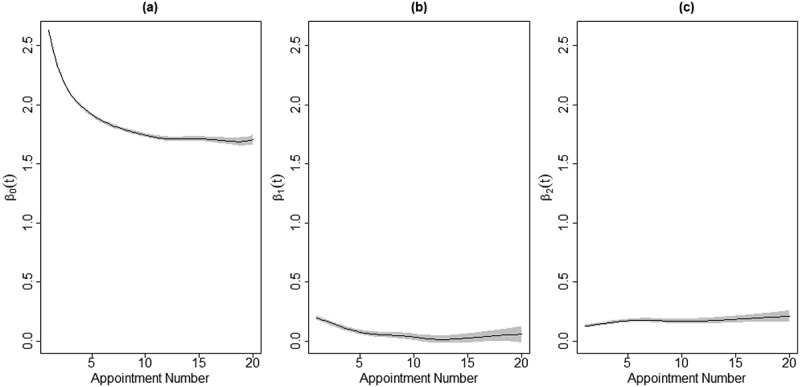
The estimated coefficient functions for the time-varying effects of SDS SI and SA on client distress for clients who presented above the high-cut for distress

The estimated coefficient functions according to Model (1) are presented in Figure 4. As in the linear model using all clients, we can see from the intercept coefficient function that average distress among noSI_noSA Group clients improves greatly, particularly within the first 12 appointments. The average contribution to overall distress from SDS SI also diminishes, especially within the first 7-10 appointments. Unlike in the model with all clients, the additional distress experienced by clients with SA does not appear to improve throughout treatment and even appears to worsen, though by less than a tenth of a point on average.

**Figure 5.**
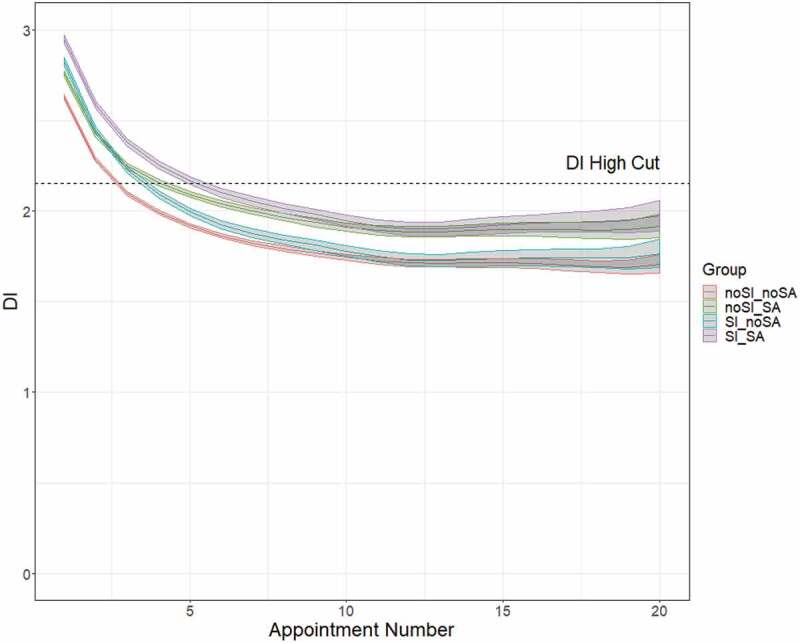
Predicted DI and the associated prediction bands for the four client groups among students who initially presented above the high-cut for distress

Figure 5 shows the predicted distress levels for the linear TVEM. Since these data included only students who began treatment with high levels of distress, all groups—including those who endorsed neither past SA nor SDS SI within the past 2 weeks—had high initial predicted distress. The noSI_noSA Group has a slightly lower predicted distress at the first appointment than that of any other group, but the difference between their initial stress and the initial distress predicted for the other groups is far more subtle than it was when the model was fit to the unrestricted data. Encouragingly, all four groups are predicted to see enough reduction in their distress that by their 6^th^ appointment, clients report levels of distress below the 2.15 threshold.

**Figure 6.**
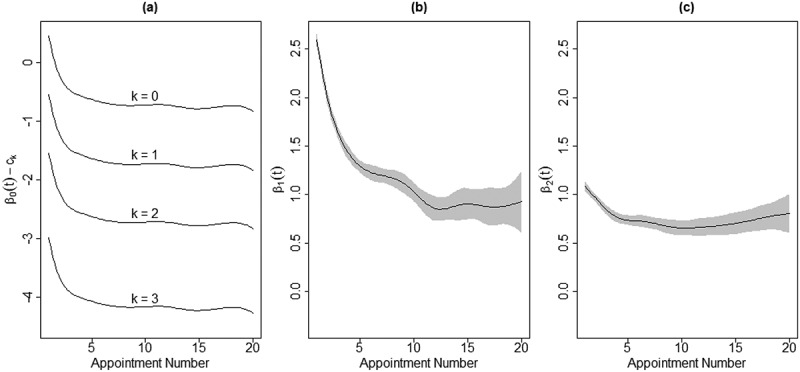
The estimated coefficient functions for the time-varying effects of SDS SI and SA on client CCAPS SI for clients who presented above the high-cut for distress

Fitting Model (2) to the restricted sample yields much the same results as when the larger data set was used. Figure 6 shows the estimated coefficient functions, which look very similar to the estimated coefficient functions displayed in Figure 3, when all clients were used in fitting the model. It is clear again that clients with SDS SI have a much higher log odds ratio of higher CCAPS SI scores than do clients without SDS SI, but that this difference is greatly attenuated throughout treatment. The greatest reduction in the effect of SDS SI at the first appointment on CCAPS SI score occurs within the first five appointments, and decelerated improvement continues until around the 12^th^ appointment. Also, as in the model with all clients, $\beta_{2}\left(t\right)$ has a much gentler slope than $\beta_{1}\left(t\right)$. This indicates that, while the effects of SA are less pronounced than those of SI to begin with, those effects see far less improvement on average and, in particular, very little if any improvement beyond the fifth appointment.

### Rate of change in distress – high-distress students

Finally, the analyses described above for the second hypothesis were repeated on a subsample of students who began treatment with a high level of distress (i.e. DI greater than 2.15). It was hypothesized that, even for this more acutely distress sample, students reporting a history of SA and SI within the past 2 weeks at their first appointments would see a slower rate of improvement in their levels of distress, therefore requiring more sessions to see improvement. Once again, this hypothesis is refuted by the estimates from Model (1). Instead, the students with both SA and SI see about the same or more improvement in distress as the other groups do over a given period of time.

Figure 5 shows how distress changes throughout treatment for each client group. For all four groups, reduction in distress is predicted to be most rapid during the first three appointments, with milder improvement predicted until around the 15^th^ appointment. Beyond the 15^th^ appointment, improvement appears to plateau across all four groups, and subtle differences in their trajectories are clouded by wider confidence bands due to a dearth of data for clients in such high-numbered appointments. Thus, all four groups are very similar in their rates of change. For example, the average reduction in DI from session 1 to session 5 was 0.71 for the noSI_noSA group, 0.83 for the SI_noSA group, 0.66 for the noSI_SA group, and 0.78 for the SI_SA group. Therefore, clients presenting at the first appointment with both a history of SA and recent SI improved in distress more rapidly than those with neither SI nor SA and those with only SI but not SA.

## Discussion

The findings from this study begin to capture how levels of distress (as a global indicator of treatment effects) and SI change over the course of treatment for students who present to treatment at UCCs with varying suicide risk and those without suicide risk. Consistent with results of previous studies that found routine treatment at UCCs led to reductions in symptoms of depression, anxiety, social anxiety, eating disorders (A.A. McAleavey et al., 2019) and general distress (Center for Collegiate Mental Health (CCMH), 2017), this study found that overall treatment at UCCs reduced some level of distress and SI among all students regardless of risk or initial level of distress, with the greatest success evidenced in the first five appointments. These results were also consistent with the study hypothesis that routine treatment would be associated with some decreases in distress and SI among those who reported current SI with no history of SA, no current SI with history of SA, and current SI with history of SA.

It was also hypothesized that students reporting a history of SA and current SI (i.e. those with a more chronic symptom presentation) at their initial appointment would demonstrate a slower decrease in distress than the other groups. Interestingly, while students presenting with current SI and a history of SA demonstrate great initial improvements in distress and SI, their prognosis appears to be limited over the course of treatment. For students who present to UCC’s with current SI, distress appears be greatly reduced within 10-12 sessions. However, for students with current SI and a history of SA, the improvements in distress are limited after 10-12 sessions. Among students who enter treatment with high levels of distress, improvements in distress appear to plateau across all four groups after the 15^th^ session. Particularly, distress among students with a history of SA, who present to treatment above high cut distress, shows limited improvement throughout treatment.

While all groups of students demonstrate a reduction in SI as a result of treatment, improvements among students with history of SA seem to plateau earlier in treatment than among students in other groups. Among students who present to treatment with high levels of distress and current SI, the greatest improvement seems to happen in the first five sessions with some improvement continuing until approximately appointment 12. Improvement in SI for individuals with history of SA who present to treatment with high levels of initial distress seems to be limited to the first five sessions and does not occur to the same extent as for individuals without history of SA.

One important caveat when interpreting these results is that they cannot necessarily be taken causally, being based on observational data only. For example, the lack of improvement in distress and SI beyond a certain number of sessions might not necessarily be caused by ineffective therapy. Instead, it is entirely possible that the students who attend, say, 15 sessions of treatment, are still attending precisely because their distress or SI is still not subsiding (i.e. reverse causation). Thus, while students who present with history of SA still see large initial improvement (e.g. within 4-5 sessions), their average distress and SI do not decrease to as low of levels as other students’ do in response to treatment. Consequently, students presenting with history of SA may be less responsive to treatment, in general, and may need more or specialized treatment. Arbitrary session limits for short-term counseling (e.g. 4-5 sessions) may not be appropriate for students with a history of SA whose levels of risk and the chronicity of symptoms may be better managed with longer-term treatment.

Collectively, these findings offer a number of implications for the assessment and treatment of students presenting with higher levels of distress and suicide risk at UCCs. First, based on study findings, the assessment of both SI and SA history among students entering treatment may be important. These variables, especially history of SA, may account for heightened levels of distress and, in some cases, less responsiveness to treatment-as-usual. Thus, assessment of these variables at the initial session may inform the trajectory of and plan for treatment. Further, if the average length of treatment-as-usual at UCCs is approximately five sessions, then students requiring 20 or more sessions seem outside of the scope of care at UCCs. Thus, UCCs may need to arrange for longer-term care to effectively manage the risk represented by SI and SA.

Second, apparent limits to improvement in response to UCC treatment among individuals with current SI and a history of suicide attempts suggest that treatment-as-usual at UCCs may not address SA severity or chronicity (i.e. the risk of repeated engagement in suicidal behaviors is high) and that some students need care beyond 15 sessions. In fact, the post-discharge suicide rate for individuals who have been hospitalized with suicidal thoughts or behaviors is nearly 200 times the global rate during the first 3 months after discharge and, even years after discharge, rates for these individuals remain high for many years after discharge (Chung et al., 2017). Thus, students presenting to UCCs with current SI and a history of SA may require additional services beyond the scope of routine practice at UCCs, and arbitrary session limits on this group would be ineffective. Additional services may target the function of chronic SI and behaviors, or distress related to previous attempts.

Research suggests that suicide functions as a means of escaping from unmanageable emotional distress (Baumeister, 1990). SI, in particular, has been linked with emotional dysregulation (Rajappa et al., 2012; Weinberg & Klonsky, 2009) as well as the inability to flexibly respond to and manage negative emotions (Carpenter & Trull, 2013). Thus, UCCs may consider offering specific treatments to address these concerns among students who present with current SI and history of SA. For example, DBT is one such treatment that emphasizes distress tolerance and emotion regulation (Linehan, 1993) and has been shown to be an effective treatment for suicidal college students (Pistorello et al., 2012). Future research may focus on testing empirically validated treatments among groups of students with varying levels of suicidal risk (e.g. a group with current SI and no history of SA, a group with current SI with history of SA) to see whether effects of treatment vary for different groups.

Finally, these results offer implications regarding session limits and allocation of resources for students presenting with risk at UCCs. Specifically, findings suggest that improvements in distress and SI seem to taper off after five sessions, especially for students with current SI but no history of SA. However, results suggest that students with history of SA may need further care beyond what session limits allow. UCCs face pressure to reduce treatment length, serve more students, manage suicide risk, and reduce distress. The findings from this study highlight a contradiction in these aspirations. A key result of this study suggests that students with more complex psychiatric histories require more treatment than is typically provided as UCCs implement session limits. Arbitrary session limits to increase a center’s capacity to serve more students with less treatment are likely to prove ineffective for students presenting with greater levels of risk and distress (i.e. students with SI and history of SA). Thus, while UCCs may have limits to the number of sessions offered to students with SI without SA, they may consider increasing the number of sessions offered to students with a history of SA or referrals out to community providers once session limits are reached by these students. However, UCCs may not be equipped with resources and funding to provide more treatment to the subset of students who require it. With that said, further research is needed to determine which types of treatment and at what dosage will most benefit students at greatest risk of suicide to inform the discussion about allocation of resources and funding.

Despite the strengths of this study, results must be considered in the context of limitations. First, this study used self-report measures of independent and dependent variables. Future studies may consider the use of objective measures of SI and DI. Another limitation of this study is that fewer data points existed for higher appointments, making data for these appointments less representative. As a result, drawing reliable conclusions about distress levels and SI at high appointment numbers is difficult. For example, relatively few clients attended 17, 18, 19 or 20 appointments. Thus, the margins of error (and corresponding 95% confidence bands) are wider at these appointments. A third limitation of this study is the rather restrictive proportional odds assumption imposed by Model (2). The proportional odds assumption is convenient mathematically and simplifies interpretation of the regression coefficient functions but can be overly restrictive because it implies a constant relationship for the logit of each level of the ordinal response variable. For example, it implies that SI and SA have the same impact on the log odds of a SI score above 1 as they do on the log odds of reporting a SI score above a 3. While this is possible, it is not necessarily the case. Future work could aim to extend the methodology used here to fit other ordinal regression models with less restrictive assumptions.

One final point worth noting is that all of the estimated models described above have relatively low predictive power, as seen by the relatively low proportions of deviance explained, reported in Table 2. This is to be expected given the complexity of and inherent variability in measuring human distress. Regardless, low predictive power does not detract from the ability to effectively estimate the relationships we are interested in.

The non-parametric models upon which this study’s analyses were based are less widely used in the context of studying treatment outcomes, though their use is becoming more widespread. The novelty of this approach being used in the context of analyzing treatment outcomes over time may be considered a strength of this study. Nonparametric models offer opportunities to analyze the relationship between treatment outcomes (i.e. levels of distress and SI) and number of sessions that parametric tests may not be able to capture.

Still, while the benefits of nonparametric approaches like TVEM were touted above, the name nonparametric is a bit of a misnomer in that these models still depend on estimating parameters and other user-specific choices. In particular, researchers must decide on the appropriate number of basis functions (though, as noted above, there is reason to believe that the precise number is not as important as choosing a large enough basis) and smoothing parameters, which determine the degree of smoothing to apply to the estimated functions, must be estimated. Thus, while nonparametric methods aim to let the data speak for themselves, they do not free researchers from all burden in statistical modeling. In addition, nonparametric methods come with another cost: they require larger datasets than the usual parametric methods to achieve the same level of accuracy in estimation. This limitation did not pose a problem for the current study due to the large sample size, but future researchers interested in using TVEM should keep this in mind.

Furthermore, TVEM is merely one example of a very broad class of nonparametric methods that enable the study of time-varying effects. In this paper, a TVEM was used to study the time-varying effects of static (non-time-varying) variables, like SI and SA, on DI and SI. This model could also incorporate time-varying independent variables, such as level of depression on the CCAPS depression subscale at each session, assuming that depression level at session *t* is only related to the dependent variable at the same time—what is referred to as the functional concurrent model in FDA literature (Ramsay & Silverman, 2005). However, one could imagine that both current and past values of depression relate to current distress or current levels of suicidality. This is an example of what is known as a historical functional linear model (Malfait & Ramsay, 2003; Morris, 2015). In addition, as was discussed above, a more complex random effects structure should be considered, though doing so may first require additional software development. Future research will need to continue to explore these and other nonparametric models to better understand the complexities of suicidality and inform treatment.

## Supplementary Material

Supplemental Material

## Data Availability

The data reported in this manuscript were obtained from Center for Collegiate Mental Health. http://ccmh.psu.edu/available-data

## Note

1. We used the R computing software to perform the analyses in this paper, in particular the pffr package. The framework on which this package is based theoretically enables multiple partially crossed random effects (Scheipl et al., 2015), however the current implementation of the package does not allow for this with longitudinal data (i.e. *sparse functional data*, in functional data parlance). A fairly new tvem package in R also implements TVEM but does not currently allow for any random effects or hierarchical structure.
