# Supplementary material for: A comparison of treatment response between college students with and without suicide risk at university counseling centers
Source: Cogent Ment Health. 2023 Apr 17;2(1):2169583. doi: 10.1080/28324765.2023.2169583 (PMC12443023; doi:10.1080/28324765.2023.2169583)

**Supplementary Material**

**A Comparison of Treatment Response Between College Students with and without Suicide Risk at University Counseling Centers**

***Exploratory Data Analysis***

Before turning to a statistical model, exploratory analysis was performed on the data to inform our modeling decisions. In order to assess whether and how client distress changes throughout treatment, one natural starting point is to consider the distribution of distress among clients at their first appointment compared to the distribution of distress at clients’ final appointment. Figure 1 facilitates such a comparison by overlaying density curves of client distress (DI) across all first and last appointments, respectively. When comparing density curves, the shift left-ward—from a greater concentration around higher values of distress at the first appointment to lower values of distress at the last appointment—is striking, affirming the effectiveness of treatment. However, while encouraging, the practical implications of Figure 1 are limited by the fact that the last appointment is relative to the client (e.g. the last appointment for a client could be only the client’s second appointment, or it could be their 50^th^).

This concern is ameliorated in Figure 2 by comparing density curves of distress at clients’ first, fifth, and tenth appointments. In addition to corroborating the message from Figure 1, Figure 2 adds incrementality to the conclusion, hinting at the rate of change in distress over appointments regardless of length of treatment, time of semester, other treatments received, counseling center policies, treatment limits, etc. For instance, it is clear from the left-ward shift in concentration of DI from appointment 1 to appointment 5 that a great deal of distress is reduced in these first five appointments. However, equally interesting is the deceleration of change in the distribution of distress from appointment 5 to appointment 10. In these latter five appointments, it seems that relatively little progress was made in relieving client distress compared to the first five appointments of treatment. Overall, this reflects a flattening of the change curve at session 10 but does not adequately illustrate the variation in rate of improvement for students receiving longer courses of treatment.

These insights provide a useful starting point but lack granularity. To better understand the trend in typical client distress throughout treatment, we inspected the within-appointment mean distress (i.e. the average distress of all clients at a particular appointment number) over 20 appointments, as illustrated in Figure 3. Here, it becomes clear that the reduction in distress was rapid but waning in the first 5-6 appointments, seemingly reaching a plateau thereafter. Taken together, Figures 1 through 3 provide evidence of marked early improvement in distress among the first five or so appointments, followed by a flattening of the change curve. However, these descriptions apply generally to all clients in the data and fail to capture any client-specific differences or differences between the four groups of interest.

Finally, Figure 4 goes one step further, providing the within-appointment mean distress across the four groups of clients described above. At a cursory glance, a similar pattern of improvement in distress can be found within each of the four groups, characterized by early improvement that is followed by a plateau, reached typically around the sixth or seventh appointment. Interestingly, while the SI_noSA Group begins at a slightly higher level of distress than the noSI_SA Group, the two seem to level off at about the same level of distress. Even the SI_SA Group seems to end up with nearly the same, if not a slightly higher, level of distress, although this is somewhat obscured by the heightened uncertainty in later appointments (attributable to fewer clients having an 18^th^, 19^th^, or 20^th^ appointment). Keeping these findings in mind, the time-varying effect model used in the main article was chosen to explore differences between these four groups while simultaneously accounting for client-specific differences.

***Additional Models: Accounting for Treatment Length***

Models (1) and (2) in the main article included SI and SA as the only independent variables. However, we also considered accounting for a client’s length of treatment as a possible confounding variable. To do this, we added as another control variable to both models the number of sessions a client attended in total. Adding this term, Model (1) becomes

|  | $DI_{ij}\left( t_{ij} \right)=\beta_{0}\left( t_{ij} \right)+\beta_{1}\left( t_{ij} \right)SI_{i}+\beta_{2}\left( t_{ij} \right)SA_{i}+\beta_{3}\left( t_{ij} \right)(\#sessions)+b_{i}(t_{ij})+\varepsilon_{ij}(t_{ij})$, | (3) |
| --- | --- | --- |

where $\beta_{3}\left( t_{\mathrm{ij}} \right)$ represents the shift in mean level of DI at appointment $j$ for a client $i$ who attends one additional session, holding constant whether they endorse SI or SA. In addition, $\beta_{1}\left( t_{\mathrm{ij}} \right)$ and $\beta_{2}\left( t_{\mathrm{ij}} \right)$ can now be interpreted while holding constant the number of sessions a client attended, which removes the possibility of #sessions confounding the effects of either SI or SA on DI. Similarly, Model (2) becomes

|  | $S_{ij}^{*}\left( t_{ij} \right)=\beta_{0}\left( t_{ij} \right)+\beta_{1}\left( t_{ij} \right)SI_{i}+\beta_{2}\left( t_{ij} \right)SA_{i}+\beta_{3}\left( t_{ij} \right)\left( \#sessions \right)+b_{i}\left( t_{ij} \right)+\varepsilon_{ij}\left( t_{ij} \right).$ | (4) |
| --- | --- | --- |

***Results: Accounting for Treatment Length***

Again, both Model (3) and Model (4) were applied to the two different samples described in the main article. Table 1 summarizes all four models, including the number of clients included in each model as well as the proportion of deviance explained by each model. These values are all extremely similar to the ones reported in the main article, when #sessions was not included in the models. Furthermore, Figures 5, 7, 8 and 10 show the various coefficient function estimates for each of the four models. The main point here is the effect of #sessions, which is shown in panel (d) for each plot. Note that in each case, this effect was resolutely near 0 across all appointments, indicating that the number of sessions of treatment a client has in total does not predict that client’s distress or CCAPS SI. Figures 6 and 9 are included for completion and show the predicted DI from Model (3) when applied to all clients and to just those clients who present above the high-cut for distress. Again, these are virtually unchanged from the analogous plots displayed in the main article.

| Table 1  *Sample sizes for each model and the proportion of the deviance explained by the estimated model.* | | | |
| --- | --- | --- | --- |
| Sample | Model | N  (number of students) | Deviance Explained |
| All | Linear | 101,354 | 10.5% |
|  | Ordinal | 101,354 | 7.67% |
| Above High Cut | Linear | 34,746 | 23.9% |
|  | Ordinal | 34,746 | 8.16% |

Figure 1

*Density curves of client distress (DI) across all first and last appointments*


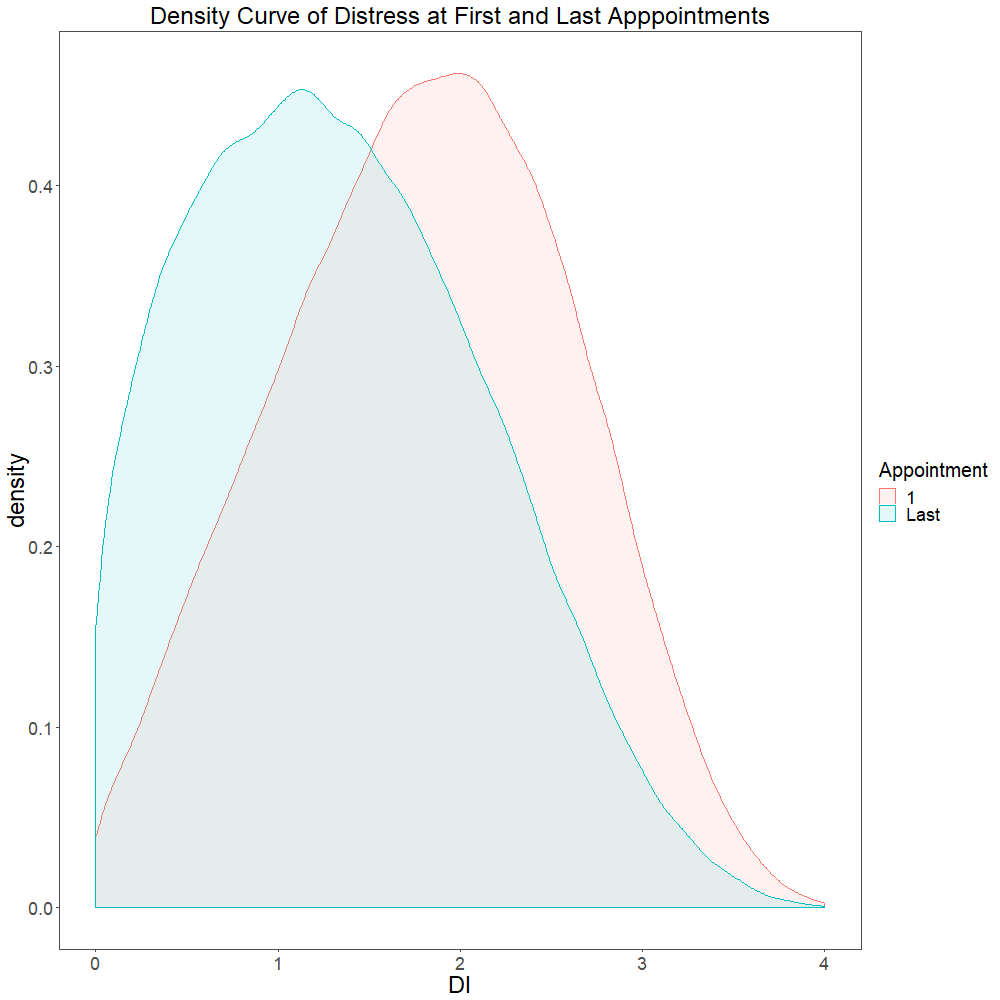


Figure 2

*Density curves of client distress (DI) across at first, fifth, and tenth appointments*


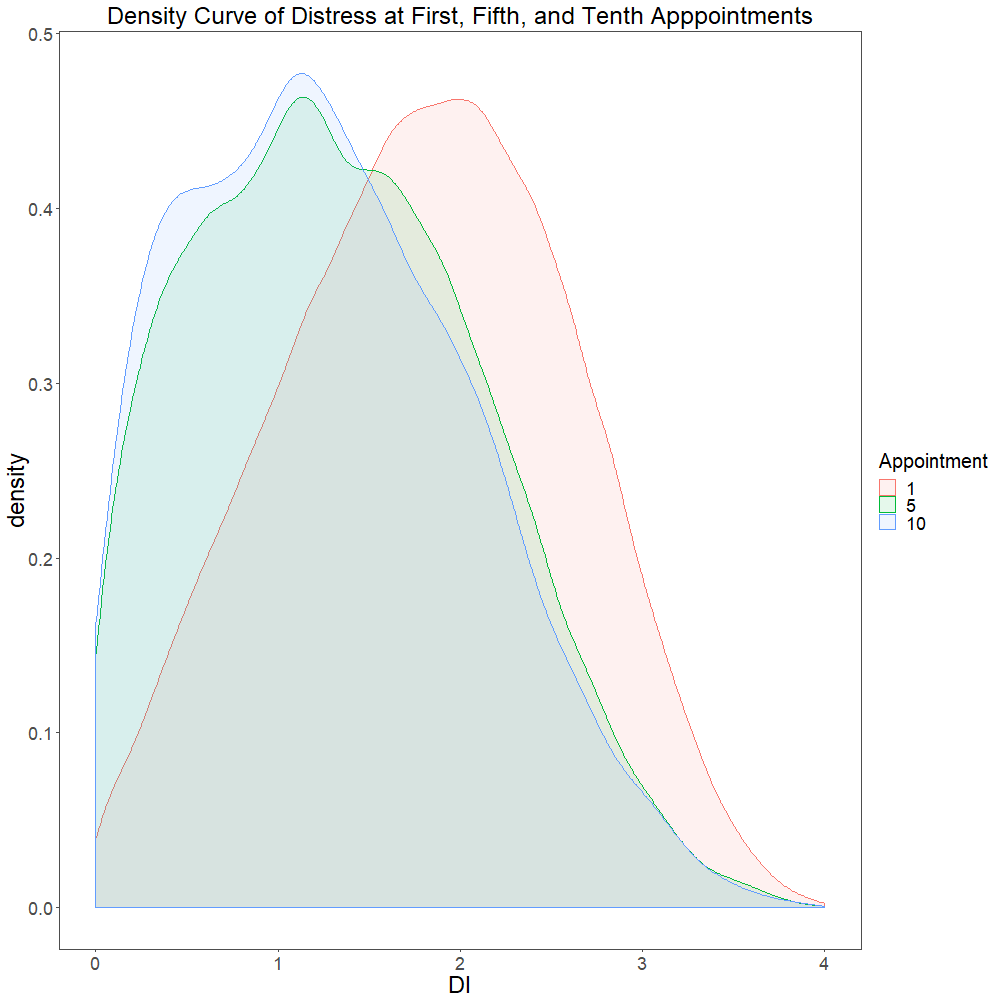


Figure 3

*Within appointment mean DI over 20 appointments*


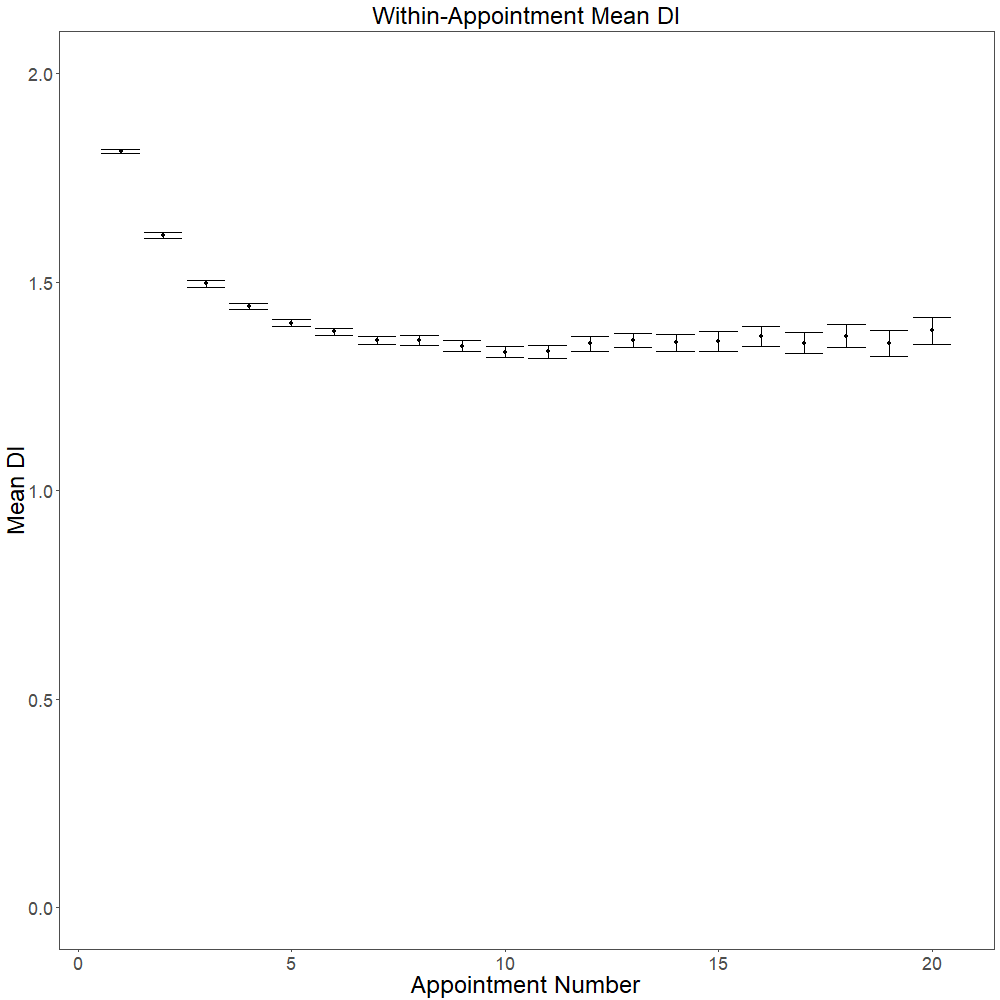


Figure 4

*Within appointment mean DI by group*


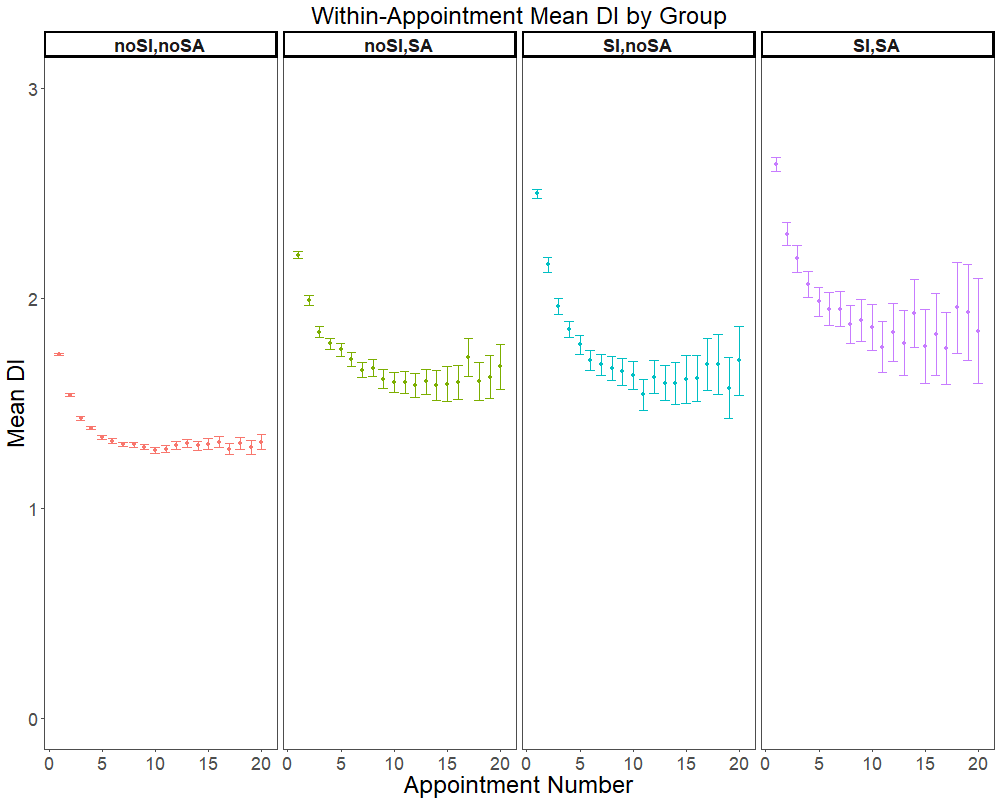


Figure 5

*The estimated coefficient functions for the time-varying intercept (a), and the time-varying effects of SDS SI (b), SA (c), and #Sessions (d) on client distress*


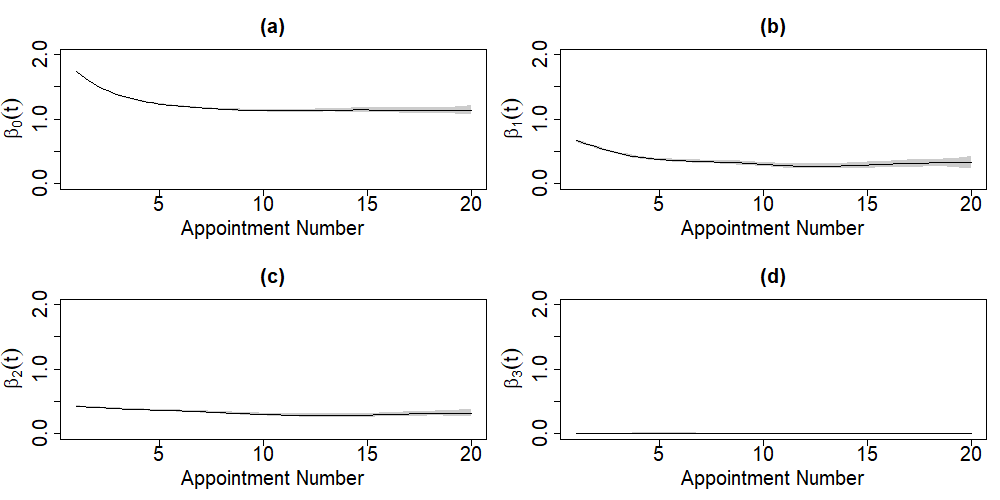


Figure 6

*Predicted DI and the associated prediction bands for the four client groups*


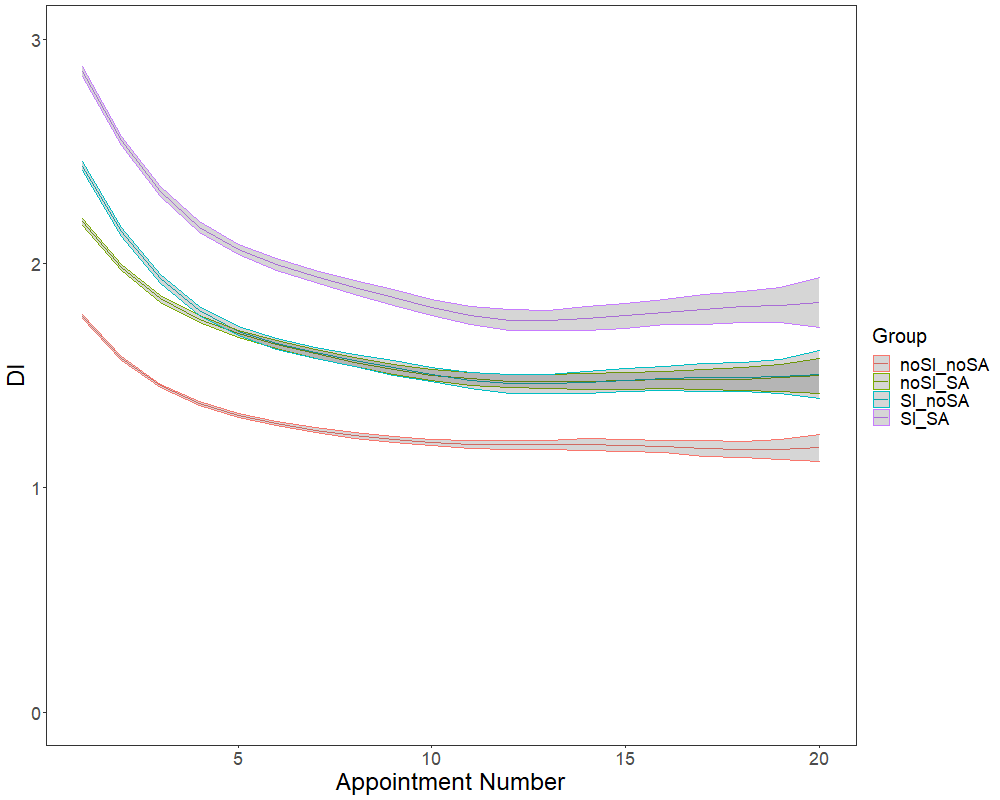


Figure 7

*The estimated coefficient functions for time-varying intercept (a), and the time-varying effects of SDS SI (b), SA (c), and #Sessions (d) on client CCAPS SI*


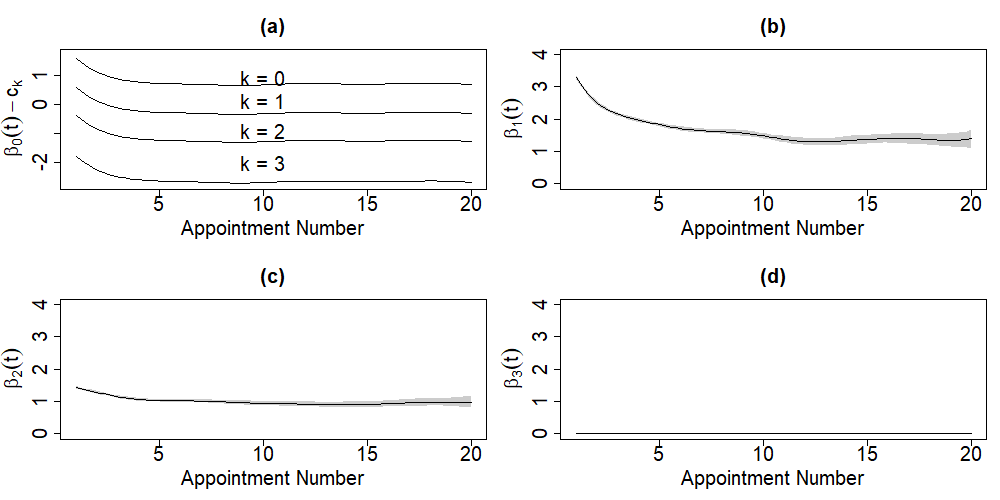


Figure 8

*The estimated coefficient functions for the time-varying intercept (a), and the time-varying effects of SDS SI (b), SA (c), and #Sessions (d) on client distress for clients who presented above the high-cut for distress*


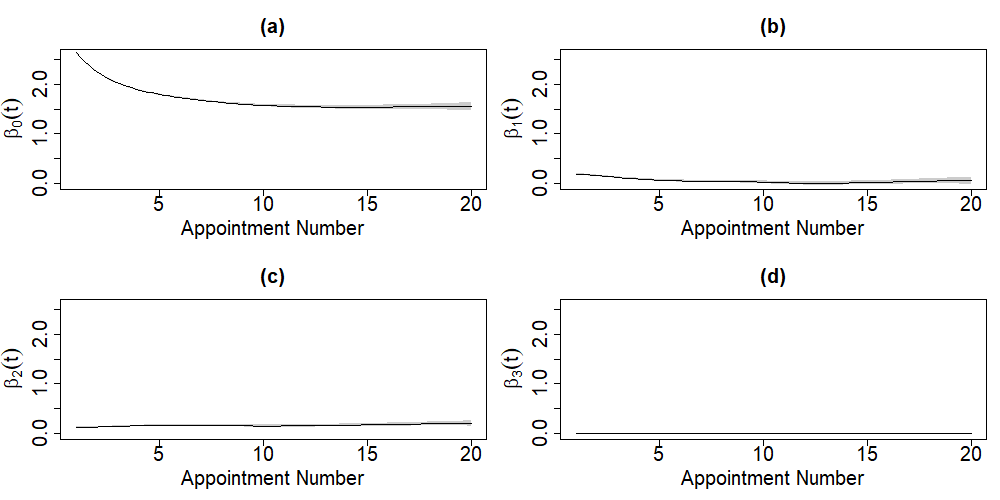


Figure 9

*Predicted DI and the associated prediction bands for the four client groups among students who initially presented above the high-cut for distress*


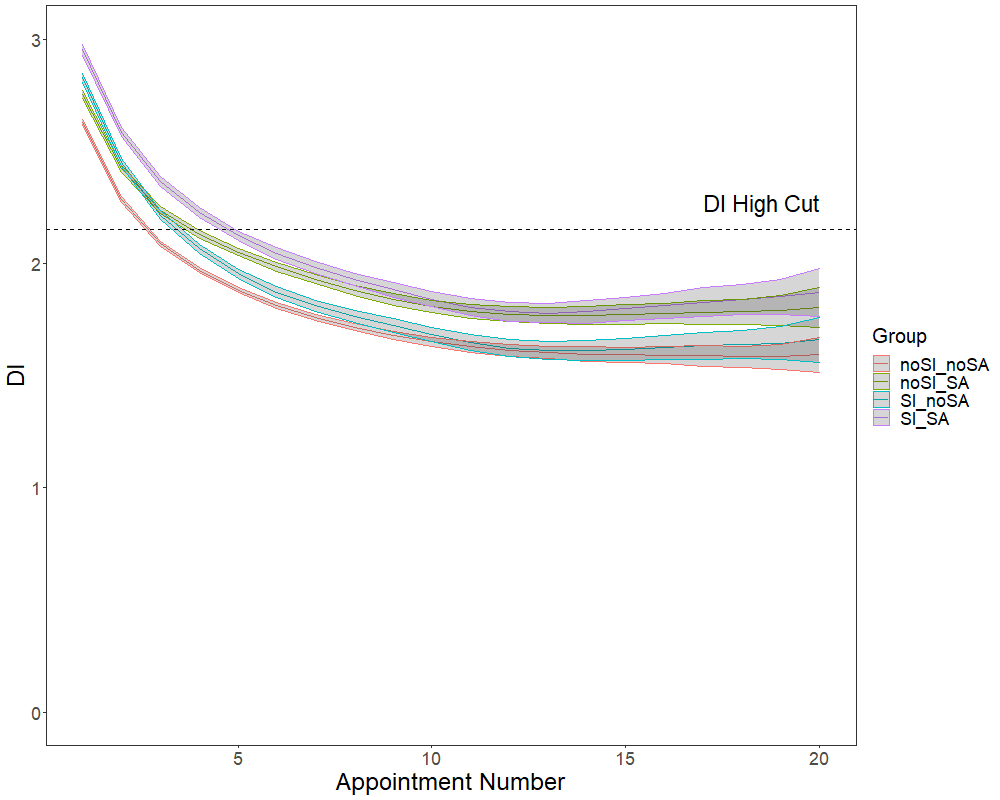


Figure 10

*The estimated coefficient functions for time-varying intercept (a), and the time-varying effects of SDS SI (b), SA (c), and #Sessions (d) on client CCAPS SI for clients who presented above the high-cut for distress*


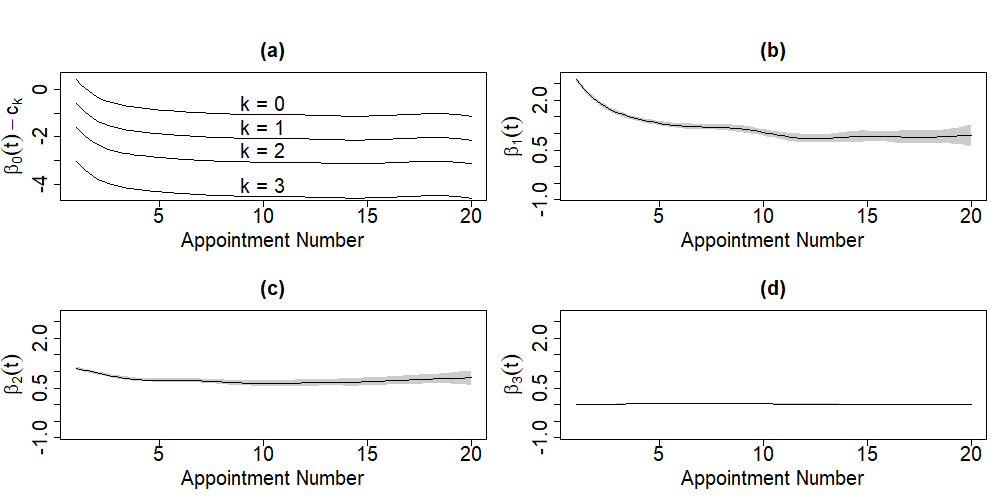

Supplement: Supplemental Material [file OAMH_A_2169583_SM6519.docx]
